# Overlap syndrome with Sjögren’s syndrome and systemic sclerosis in a steel rolling mill worker: a case report

**DOI:** 10.1186/s40557-016-0106-3

**Published:** 2016-06-02

**Authors:** Min-Kee Yi, Won-Jun Choi, Sung-Woo Han, Seng-Ho Song, Dong-Hoon Lee, Sun Young Kyung, Sang-Hwan Han

**Affiliations:** Department of Occupational & Environmental Medicine, Gachon University Gil Medical Center, Incheon, Korea; Department of Internal Medicine, Gachon University Gil Medical Center, Incheon, Korea

**Keywords:** Overlap syndrome, Sjögren's syndrome, Systemic sclerosis, Silica

## Abstract

**Background:**

There are few reports about work-related factors associated with Sjögren’s syndrome. We report a case of overlap syndrome with Sjögren’s syndrome and systemic sclerosis.

**Case presentation:**

A 54-year-old man was admitted due to dyspnea on exertion. The results of physical examination and laboratory findings were compatible with Sjögren’s syndrome with systemic sclerosis. The patient had no pre-existing autoimmune disease, and denied family history of autoimmune disease. The patient worked in the large-scale rolling department of a steel manufacturing company for 25 years. Hot rolling is a rolling process performed at between 1100 °C and 1200 °C, generating a high temperature and a large amount of fumes, involving jet-spraying of water throughout the process to remove the instantaneously generated oxide film and prevent the high generation of fumes. In this process, workers could be exposed to silica produced by thermal oxidation. Other potential toxic substances including nickel and manganese seemed to be less likely associated with the patient’s clinical manifestations.

**Conclusions:**

Occupational exposure to silica seemed to be associated with the patient’s clinical manifestations of overlap syndrome with Sjögren’s syndrome and systemic sclerosis. Although the underlying mechanism is still unclear, autoimmune disease including Sjögren’s syndrome affects women more often than men and there was no family history of autoimmune disease. These suggested that there was an association between occupational silica exposure and the disease of the patient. Future research about the association between long-term low dose exposure to silica and the development of autoimmune diseases should be encouraged.

## Background

Connective tissue diseases are systemic autoimmune diseases characterized by the appearance of a variety of symptoms and organ infiltration [1]. Although widely accepted classification standards exist for each disease, some undifferentiated connective tissue diseases are difficult to classify. Overlap syndrome is a condition in which the patient develops symptoms corresponding to two or more classification standards [2]. Although overlap syndrome can cause confusion during diagnosis, it is important because it can provide clues to the causes and pathogenic mechanisms of related diseases. Sjögren’s syndrome is an autoimmune exocrinopathy, in which systemic diseases, such as arthritis, interstitial lung disease, and kidney disease, can develop in addition to characteristic dry eye and mouth symptoms [3]. Sjögren’s syndrome occurs more frequently in female patients patients than in male patients, especially in middle-aged women. Although the prevalence rate is known to be 0.5–1 %, no accurate reports are present on the prevalence rate in Korea. Sjögren’s syndrome was first described by Johann Mikulicz in 1892 as “Mikulicz’s syndrome” after discovery of the infiltration of small, round cells into the salivary glands of a 42-year-old farmer with bilateral parotid gland enlargement. In 1933, a Swedish ophthalmologist, Henrik Sjögren, first used the term “keratoconjunctivitis sicca” to describe the dry eye symptom in 13 patients with accompanying rheumatoid arthritis among 19 patients with dry eye and mouth symptoms, differentiating it from “xerophthalmia,” the dry eye symptom due to vitamin A deficiency [4]. Although there have been some reports caused by silica, these are considered to be rare [5]. Systemic sclerosis is a disease characterized by lymphocyte deposition and fibrosis of various organs, with fibrosis developing mainly in the skin, kidney, heart, lungs, and digestive system. Lung infiltration occurs in 70 % of systemic sclerosis patients [6], and although the precise cause of the disease has not been identified, it is known that endothelial cells are damaged and fibroblasts are activated by an autoantibody-mediated immunological mechanism [7]. Here, we report the case of a steel rolling mill worker with overlapping features of Sjögren’s syndrome and systemic sclerosis.

## Case presentation

### Patient information

Fifty-four year old male

### Chief complaints

The chief complaint of the patient included breathing difficulty and coughing during exercise that developed 2 months previously.

### Current disease history

Breathing difficulty and coughing aggravated further during mountain hiking. A pulmonary disease was suspected from the findings of chest radiographs. The patient was hospitalized to obtain an accurate diagnosis and for treatment.

### Past medical history

On a special health examination performed 5 months before admission, no unusual findings were observed on the plain chest radiography. The patient had no ophthalmologic diseases, but complained of eye dryness for several months. He frequently used artificial tears because of the eye dryness.

### Personal history

The patient had a history of smoking 20 cigarettes a day for 20 years and then 10 cigarettes a day for 10 years; however, he quit smoking 3 months previously. He has a history of consuming five bottles (350 mL) of *soju* (approximately 18 % alcohol by volume) per week. No significant family history of autoimmune diseases was noted.

### Physical examination findings

On admission, the patient’s vital signs were stable with blood pressure of 140/80 mmHg, pulse rate of 86 beats/min, respiratory rate of 20 breaths/min and body temperature of 37.0 °C. Although the patient showed an acute ill-looking appearance, his consciousness was clear, cardiac sound regular, and no heart murmur was heard. Fine inspiratory crackles were heard at both lower lung fields. Finger clubbing was not observed, but fingers were swollen and skin was thickened. The findings in the abdomen, extremities, and neurological examination were normal.

### Radiological findings

**Fig. 1:**
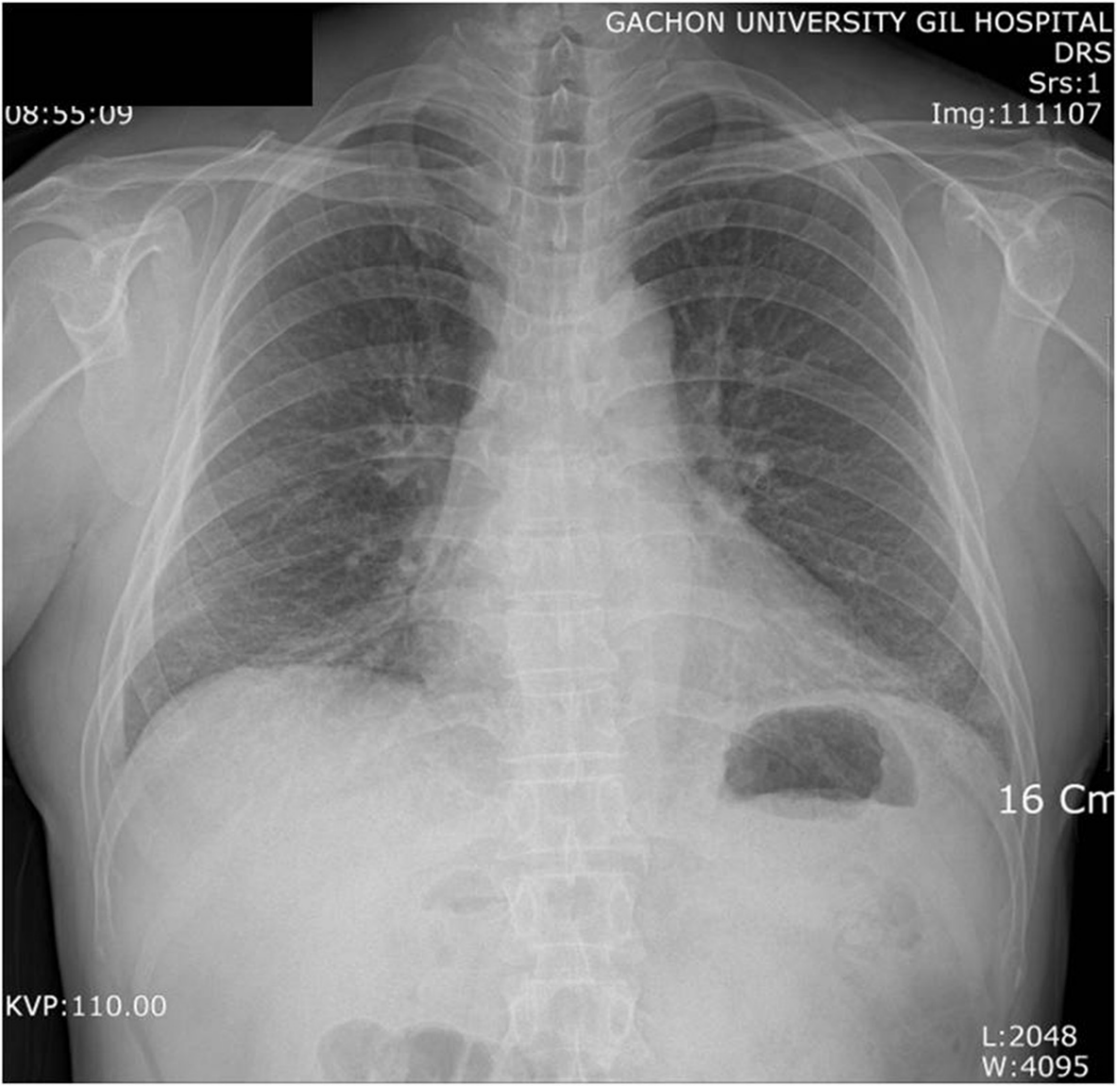
A chest radiograph. Ground glass opacities are seen in both lower lung fields

**Fig. 2:**
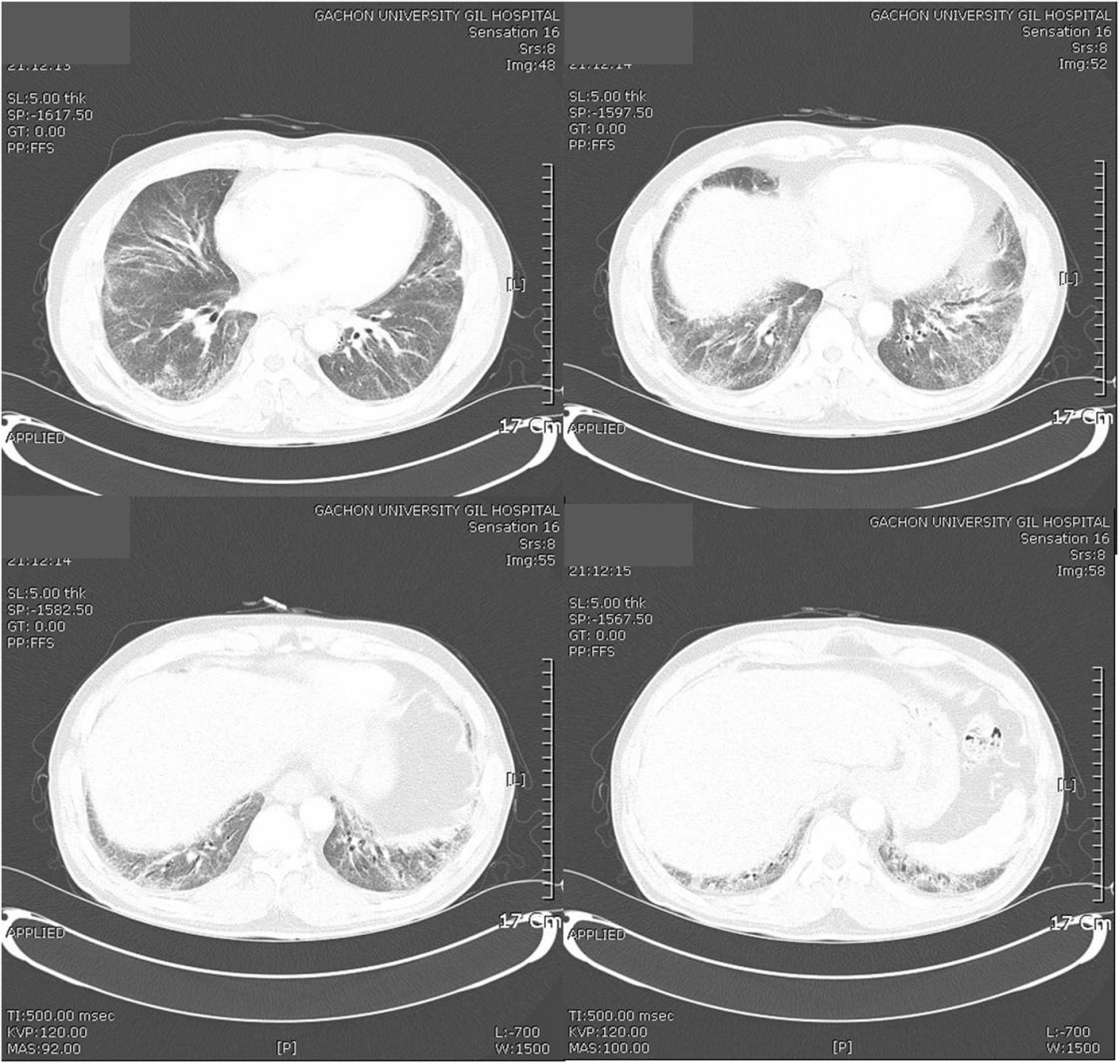
Chest computed tomography images. Peribronchial and subpleural reticulation and ground glass opacities are seen in both lungs, predonminantly in the lower parts of the lungs

In a plain chest radiograph obtained on admission, ground glass opacities (GGOs) were observed at the lower part of both lungs (Fig. 1). In a chest computed tomography image, reticulation and GGOs were observed in both lungs, leading to the diagnosis of interstitial lung disease (Fig. 2).

### Laboratory findings

In arterial blood gas analysis performed before hospitalization, pH was 7.4; PaCO_2_, 38 mmHg; PaO_2_, 94.2 mmHg; and oxygen saturation, 97.3 %. In a peripheral blood test, the leukocyte count was 8,300/μL; hemoglobin, 5.2 g/dL; and platelet count, 436,000/μL. In a lung function test, findings of restrictive lung function disorder were obtained with forced vital capacity (FVC) 2.62 L (67 % of the predicted value), forced expiratory volume in 1 s (FEV_1_) 2.31 L (80 % of the predicted value), the ratio of FEV_1_/FVC 88 %, forced expiratory flow 25–75 % 4.76 L/sec (154 % of the predicted value), and peak expiratory flow rate 8.16 L/sec (110 % of the predicted value).

### Clinical progress

The results of bronchoscopy and a lung lavage test revealed chronic inflammation with fibrosis. The results of lung biopsy were consistent with usual interstitial pneumonitis. Breathing difficulty was alleviated with systemic steroid therapy. During admission, the patient complained of dry mouth and difficulty of swallowing dry food without liquid. Immune serologic tests were performed under suspicion of autoimmune disease. The results of serologic tests were as follows; positive for antinuclear antibody (1:2560), negative for rheumatoid factor, positive for anti-SSA (Ro) antibody, negative for anti-SSB (La) antibody and positive for anti-Scl 70 antibody. The anti-ds DNA level was 7.64 IU/mL (reference level, <7.0 IU/mL). The secretory function of the salivary gland was considered to be decreased. In a salivary gland scan, the uptake increases in the parotid gland and submandibular gland were relatively normal; however, the ejection fraction of the salivary gland after stimulation decreased by more than the moderate level. Although the results of Schirmer’s test did not satisfy the diagnostic criteria for Sjögren’s syndrome, with the right eye showing a result of 12 mm in 5 min and the left eye showing a result of 7 mm in 5 min, they were found to be lower than the normal range. Other diseases such as hepatitis C, acquired immunodeficiency syndrome, lymphoma and sarcoidosis were excluded by medical history and the results of relevant laboratory tests. Clinical findings including ocular symptom of dry eyes, oral symptom of difficulty in swallowing dry food without liquid, oral sign of abnormal salivary scintigraphy and positive autoantibody of anti-SSA (Ro) antibody were compatible with diagnostic criteria for Sjögren’s syndrome (Table 1) [8]. In addition, there were some features of systemic sclerosis (puffy fingers, interstitial lung disease and positive for systemic sclerosis related autoantibody [anti-Scl 70]) according to the revised classification criteria of systemic sclerosis (Table 2) [9]. The patient has been followed for the overlap syndrome with Sjögren’s syndrome and systemic sclerosis.

**Table 1:** Diagnostic criteria for Sjögren’s syndrome (American-European Consensus for Sjögren’s syndrome [8])

Criteria^a^	
I. Ocular symptoms (at least one)	
1. Dry eyes for at least 3 months	
2. A foreign body sensation in the eyes	
3. Use of artificial tears three or more times per day	
II. Oral symptoms (at least one)	
1. Dry mouth for at least 3 months	
2. Recurrent or persistently swollen salivary glands	
3. Need for liquids to swallow dry foods	
III. Ocular signs (at least one)	
1. Abnormal Schimer’s test (5 mm or less in 5 min)	
2. Positive vital dye staining (van Bijsterveld score 4 or higher)	
IV. Histopathology	
1. Lip biopsy showing focal lymphocytic sialoadenitis (focus score ≥1 per 4 mm^2^)	
V. Oral signs (at least one)	
1. Unstimulated whole salivary flow (1.5 ml or less in 15 min)	
2. Abnormal parotid sialography	
3. Abnormal salivary scintigraphy	
VI. Autoantibodies (at least one)	
1. Anti-SSA (Ro) or anti-SSB (La) or both	
VII. Exclusion criteria	
1. Past head and neck radiation treatment	
2. Hepatitis C infection	
3. Acquired immunodeficiency syndrome	
4. Pre-existing lymphoma	
5. Sarcoidosis	
6. Graft versus host disease	
7. Current use of anticholinergic drugs	

^a^For primary Sjögren’s syndrome, (i) any 4 of the 6 criteria, must include either item IV (histopathology) or VI (autoantibodies) or (ii) any 3 of the 4 objective criteria (III, IV, V, VI); for secondary Sjögren’s syndrome, in patients with another well-defined major connective tissue disease, the presence of one symptom (I or II) plus 2 of the 3 objective criteria (III, IV and V)

**Table 2:** Diagnostic criteria for systemic sclerosis (American College of Rheumatology/European League Against Rheumatism criteria for the classification of systemic sclerosis [9])

Item	Sub-items	Weight/score^a^
Skin thickening of the fingers of both hands extending proximal to the metacarpophalangeal joints (sufficient criteria)	-	9
Skin thickening of the fingers (only count the higher score)	Puffy fingers Sclerodactly of the fingers (distal to the metacarpophalangeal joints but proximal to the proximal interphalangeal joints)	2 4
Fingertip lesions (only count the higher score)	Distal tip ulcers Fingertip pitting scars	2 3
Telangiectasia	-	2
Abnormal nailfold capillaries	-	2
Pulmonary arterial hypertension and/or interstitial lung disease (maximum score is 2)	Pulmonary arterial hypertension Interstitial lung disease	2 2
Raynaud’s phenomenon	-	3
Systemic sclerosis-related autoantibodies	Anticentromere Anti-topoisomerase I (anti-Scl 70) Anti-RNA polymerase III	3

^a^The total score is determined by adding the maximum weight (score) in each category. Patients with a total score of 9 or higher are classified as having definite scleroderma

### Occupational history and working environment

The patient had worked for 25 years as a worker in the large-scale rolling department of a steel-manufacturing company. He was in charge of maintenance and repairs for the roughing mill and intermediate mill. His major tasks were the management of oils, roll exchange of the rolling mill for different sizes and stand-seating manufacture. Rolling is a plastic process in which the cross-sectional thickness of a material is reduced by successively pressing while passing it between two rollers. A roughing mill is the first process where slabs coming out of the furnace are rolled, and this produces dust containing silica. Hot rolling is a rolling process performed at a temperature of between 1100 °C and 1200 °C. A large amount of fumes develops at that high temperature. Generally, the hot rolling process is a wet process. As it progresses, oxidized film on the surface of the rolled material is removed and water is jet-sprayed to prevent the high generation of fumes. The final product is a scroll-shaped intermediate processed product called a coil, after which additional processing is performed in the cold rolling process. The large-scale milling process in which he was involved is a series of operations comprising the furnace, roughing mill, intermediate mill, finishing mill and cutting. The roll was being exchanged for different sizes more than once a day, depending on the work volume. To meet the production standards when changing the roll, the process of resting the new roll on a stand, dissembling the existing roll and performing the exchange was mainly performed near the rolling mill owing to the nature of the work. According to the report of working environment measurement, noise, dust, toxic metals and heat were identified as occupational hazards. In general, workers in the milling process take 30-min rest after 30-min working because of the high-heated working condition. The patient has worked on the three-shift system. The total ventilation system was applied in the plant, and the patient has regularly used personal protective equipment including mask and ear plugs.

It was verified that standard steel products produced by a series of hot rolling processes contain a maximum of 0.4 % silicon [10]. However, the concentration of free silica in the air was not measured directly. Instead, type 2 dust, which contains silicon dioxide up to 30 %, was measured as mineral dust. According to the report of working environment measurement in 2008, the concentration of type 2 dust was 1.444 mg/m^3^ and 0.417 mg/m^3^ in the roughing and intermediate mills, respectively (Table 1). Potential toxic substances other than silica were found in the milling process such as nickel, chromium and manganese, but the ambient concentration was very low or not detectable. For example, the highest concentration of nickel and manganese were measured at the roughing mill site, which were 0.0026 mg/m^3^ and 0.0051 mg/m^3^, respectively (Table 3).

**Table 3:** The results of working environment measurement for dusts in rolling mill part

Survey year	Type of dust	Site	Concentration (mg/m^3^)	Exposure limit (mg/m^3^)
2008	Type 2 dust^a^	Roughing mill	1.444	5
		Intermediate mill	0.417	
		Finishing mill	0.556	
2013	Unspecified mineral dust^b^	Roughing mill	1.349	10
		Intermediate mill	0.841	
		Finishing mill	2.384	

^a^Type 2 dust contains silicon dioxide up to 30 %

^b^Unspecified mineral dust contains silicon dioxide up to 1 %

## Discussion

The current case satisfied the diagnostic criteria for Sjögren’s syndrome owing to the conditions of ocular symptoms (dry eyes for several months), oral symptoms (need liquids to swallow dry foods), salivary gland infiltration (abnormal salivary scintigraphy) and presence of anti-SSA (Ro) antibody in the serum. Diagnosis of systemic sclerosis is based on the scoring system revised in 2013 by the American college of rheumatology and the European league against rheumatism. Patients with a total score of 9 or higher are classified as having definite scleroderma. In this case, the patient had puffy fingers, interstitial lung disease and anti-Scl 70 antibody. The patient had clinical findings compatible with systemic sclerosis although the total score of 7 was insufficient to classify as having definite systemic sclerosis. Actually, the nailfold capillaroscopy was performed at other medical institute and the result was abnormal. If there were abnormal findings in nailfold capillaries, definite diagnosis of systemic sclerosis would be possible with total score of 9. Unfortunately, we could not obtain the figures of the nailfold capillaroscopy, but only the written interpretations.

### Silica exposure and autoimmune diseases in the previous literature

The association between silica exposure and autoimmune diseases was first reported by Caplan in 1953. It was proposed with regard to the characteristic chest radiographic findings in miners with rheumatoid arthritis [11]. Later, it was called Caplan’s syndrome in several reports, including from Korea [12, 13]. Systemic sclerosis was first described by Erasmus in 1957, after which additional reports described it as Erasmus syndrome [14, 15]. Systemic lupus erythematosus (SLE) was reported by Conrad in 1996 in a uranium mine cohort study, and Yamazaki reported a case in 2007 in which short exposure for a few months caused SLE in miners [16, 17]. In a prospective study by Sanchez-Roman in 1993 on 50 workers exposed to silica, systemic diseases were found in 64 % of the workers (Table 4), among which three cases of Sjögren’s syndrome were diagnosed as primary Sjögren’s syndrome [18]. Later, five additional cases were reported, and the disease was considered to be rare [5, 19, 20].

**Table 4:** Studies on occupational silica dust exposure and primary Sjögren's syndrome

Author	Study	Publication year	Number of case	Sex/Age	Occupation	Working period
Sanchez-Roman et al. [18]	Cohort 50 factory workers (88 % female)	1993	3	6 men and 44 women	Production of scouring powder	6.1 years (mean)
Puisieux F et al. [19]	Case-report	1994	3	-	Coal miners	-
Astudillo L et al. [5]	Case-report	2003	1	M/72	Dental technician	46 years
Ferreira PG et al. [20]	Case-report	2014	1	M/75	Welder (sandblasting)	35 years

### Association between silica exposure and autoimmune diseases

Many cases of autoimmune diseases caused by exposure to silica have been identified during testing or treatment for silicosis. It is still unclear whether silicosis is a simple marker of silica exposure or presents the pathogenesis of autoimmune diseases. When silica particles enter the respiratory system, they are phagocytized by alveolar macrophages and activate inflammatory responses and fibroblasts [21]. Silica is cytotoxic, and silica particles cannot be degraded by the lysosomal enzymes of macrophages. It induces fibrosis through immune activation and chronic stimulation of macrophages leading to silicosis, and can be transported to other organs including the lymph nodes, spleen and kidney [22]. In a study on rats exposed to silica, pathological changes without fibrosis were observed in the lymph nodes [23]. The effects on the lymphatic system can be explained by immune reactions related to the early exposure to silica. Silica-mediated antibody production is known to be induced after the secretion of several cytokines and fibrogenic factors with repeated mobilization and death of macrophages. In this process, inflammatory cells are activated; activated reactive oxygen is produced; tissue reactions occur in the lung parenchyma; and inflammatory mediators such as tumor necrosis factor-α, platelet derived growth factor, and interleukin(IL) are produced [24, 25]. IL-1 activates T-helper cells and promotes antibody production in B-cells [26]. Silica also activates the immune processes that produce reactive oxygen and nitrogen [27, 28]. This adjuvant effect is consistent with the hypothesis that silica causes autoimmunity and can be the cause of cell death through necrosis [29]. The mechanism by which silica exposure causes autoimmunity has been described in the literature, and it involves the repeated mobilization and death of macrophages in response to free silica entering the body through the respiratory system, resulting in the production of various cytokines and fibrogenic factors, which causes autoimmunity. Additionally, inflammatory mediators trigger the deposition of neutrophils that secret lytic enzymes, destroy the lung parenchyma, and induce proliferation of fibroblasts, thereby causing irreversible lung fibrosis, activating helper T cells, and accelerating antibody production in B-cells, which eventually results in the induction of autoimmune diseases.

The concentration of silica could not been measured directly in this study. We tried to measure the ambient concentration of silica, however, there were technical limitations and noncooperation of the worksite. However, exposure to silica could be estimated rationally. First, there were silica-containing additives such as ferrosilicon (FeSi) and silicomanganese (SiMn). FeSi is an additive used to add silicon to iron, and it is the cheapest and most effective deoxidizer and desulfurizing agent. It is classified into FeSi45, FeSi65, and FeSi75 depending on the silicon content, with FeSi75 being used the most. FeSi is added to section steel or reinforcing steel bars to increase the strength and hardness of steel. It is added to structural steel at up to 0.4–1.8 %. SiMn is used to add manganese to section steel, and it contains up to 14–25 % silicon. Silicon content of hot rolled H-beam for building structure sold in the domestic market was found to be 0.18–0.24 %, although the result differed for each specimen [10]. Second, oxidized silica can be released into the air from the surface of heated steel. The Deal-Grove model explains the thermal oxidation mechanism of silica, in which the following three processes occur: diffusion from the ambient gas layer to the oxide surface, diffusion through the oxide layer to the substrate interface, and reaction with the substrate [30]. In a 1200 °C wet process such as the hot rolling process, 0.2 μm thickness of SiO_2_ is produced per 0.1 h of exposure. Although silica was not directly measured at the workplace, the production of silica in the hot rolling process was confirmed, and actual exposure could be reasonably inferred considering the working environment of the patient.

The subjects of previous reports about the association between silica exposure and autoimmune diseases were from relatively high-level exposure occupational group such as coal miners. In this study, the patient seemed to be exposed to relatively low level of silica dust. It is unclear that long-term low-level exposure to silica causes or induces autoimmune diseases. Although the results of previous experimental study suggest that the inflammatory response in silicosis is dose dependent to silica exposure [31], there has been lack of data about the dose–response relationship between silica exposure and autoimmune diseases. Moreover, the findings about the dose–response relationship of silicosis were derived from an animal model. It seems that there may be genetic differences and susceptibility to autoimmune disease. Another study reported that silica might have both immediate and latent effects, suggesting that low-level exposure could promote the development of autoimmune diseases [32]. Further investigations about the association between silica exposure and autoimmune diseases under low exposure to silica dust should be encouraged.

There were limited evidence about the association between autoimmunity and heavy metals including nickel [33], while cobalt is known to be associated with interstitial lung disease, so-called hard metal lung disease [34]. Moreover, hard metal lung disease is associated with giant cell interstitial pneumonitis in pathologic characteristics, which is distinguished from usual interstitial pneumonitis [34]. However, the results should be interpreted with caution because there were some limitations including no direct measurement of silica concentration and uncertainty of the relationship between low dose silica exposure and the development of autoimmune diseases.

There were 36 co-workers in the rolling mill process in 2008. They could be classified into similar exposure group according to the nature of their job and the results of working environment measurement. Based on the results of regular health examination, co-workers were not suspicious having occupational diseases except noise-induced hearing loss. Considering that autoimmune disease is uncommon disease itself and affects women more frequently than men, it is hard to exclude the association between silica exposure and the patient’s clinical manifestation in this case.

## Conclusion

Occupational exposure to silica seemed to be associated with the patient’s clinical manifestations of overlap syndrome with Sjögren’s syndrome and systemic sclerosis. Although the underlying mechanism is still unclear, the facts that there were no family history of autoimmune disease and no other relevant hazardous materials associated with the patient’s clinical manifestations support there was an association between occupational silica exposure and the disease of the patient. Future research about the association between long-term low dose exposure to silica and the development of autoimmune diseases should be encouraged.
